# Nutritional and Compositional Profile of *Hypsizygus ulmarius* Fruiting Bodies as Affected by Spent Tea Leaves and Spent Coffee Grounds Supplementation

**DOI:** 10.1002/fsn3.72141

**Published:** 2026-07-23

**Authors:** Funda Atila, Mehmet Çetin, Didem Aksu

**Affiliations:** ^1^ Bergama Vocational Training School, Greenhouse Program Ege University İzmir Turkiye; ^2^ Bergama Vocational Training School, Mushroom Program Ege University İzmir Turkiye; ^3^ Central Research Testing and Analysis Laboratory Application and Research Center Ege University İzmir Turkiye

**Keywords:** amino acid profile, antioxidant capacity, composition quality, elm oyster mushroom, fatty acid profile, mineral composition

## Abstract

Post‐brewing beverage residues are increasingly explored as sustainable substrate supplements for edible mushrooms. This study investigated how spent tea leaves (STL) and spent coffee grounds (SCG), incorporated into wheat straw at 10%, 20%, and 30% (w/w), influenced the nutritional and compositional profile, antioxidant‐related responses, and Fourier transform infrared (FTIR) spectral characteristics of *Hypsizygus ulmarius* fruiting bodies. Rather than producing uniform enrichment, STL and SCG generated distinct residue‐dependent compositional patterns. STL supplementation was associated with higher protein, ash, and amino acid contents, with the 30% STL formulation yielding the highest protein (17.37 g/100 g), ash (8.87 g/100 g), and total amino acid contents (4230.17 mg/100 g). In contrast, SCG supplementation was linked to lipid‐related changes and higher concentrations of selected minerals, including P, Mg, Fe, Zn, and Cu, with crude lipid content reaching 2.50 g/100 g. Linoleic acid remained the dominant fatty acid across treatments, although residue supplementation altered the relative distribution of major fatty acids. Antioxidant‐related responses were assay‐dependent, with 20% SCG showing the highest 2,2′‐azinobis‐(3‐ethylbenzothiazoline‐6‐sulfonic acid) radical scavenging activity (ABTS) and 30% STL the highest Cupric ion reducing antioxidant capacity (CUPRAC) value. FTIR spectra supported the compositional findings by indicating treatment‐related shifts in lipid‐ and protein/chitin‐associated regions while preserving the major glucan/chitin‐based fungal matrix. Overall, STL and SCG acted as distinct substrate supplements that differentially modulated composition‐related quality attributes of *H. ulmarius*, while also supporting the value‐added utilization of brewed tea and coffee residues.

## Graphical abstract

Supplementation of wheat‐straw substrates with 10%–30% spent tea leaves (STL) or spent coffee grounds (SCG) produced residue‐specific changes in the composition of *Hypsizygus ulmarius* fruiting bodies. STL supplementation was associated with increased protein, total amino acid content, and CUPRAC values, whereas SCG supplementation was associated with increased crude lipid, selected minerals (P, Mg, Fe, Zn, and Cu), and ABTS activity. Linoleic acid remained the predominant fatty acid across all treatments.

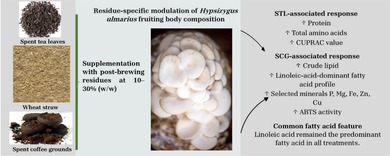

## 1 Introduction

Edible mushrooms are recognized as valuable food materials because their fruiting bodies contain proteins, essential amino acids, unsaturated fatty acids, minerals, dietary fiber‐associated polysaccharides, and phenolic‐related compounds that contribute to nutritional value and antioxidant‐associated quality traits (Łysakowska et al. 2023; Hamza et al. 2024; Losoya‐Sifuentes et al. 2025). However, mushroom composition is highly responsive to cultivation conditions, particularly substrate formulation. Because the substrate provides the nutritional basis for mycelial growth and fruiting body development, its composition may influence not only yield performance but also the nutritional and biochemical quality of the harvested product (Pellegrino et al. 2022; Atíla 2022; Luo et al. 2024).

From a food‐quality perspective, substrate‐related effects are most meaningful when they involve coordinated changes across nutritionally relevant traits rather than isolated measurements. In mushrooms, protein content alone does not fully reflect nutritional quality, since amino acid composition, especially the proportion of essential amino acids, is also important. Likewise, changes in macro‐ and micro‐element contents may alter dietary contribution, while shifts in fatty acid distribution may influence lipid‐related quality attributes even when total lipid content remains relatively low. Therefore, substrate supplementation should be evaluated through a multi‐trait assessment covering proximate composition, amino acids, minerals, fatty acids, antioxidant‐associated traits, and structural characteristics.

Post‐brewing beverage residues have attracted increasing attention as substrate supplements because they combine waste‐utilization potential with chemically relevant residual constituents. Spent tea leaves (STL) and spent coffee grounds (SCG) are particularly notable in this respect (Yang et al. 2016; Alsanad et al. 2021). Although both originate from brewed beverages, they are not compositionally equivalent. SCG may retain residual lipids, caffeine, chlorogenic acid derivatives, minerals, and nitrogen‐containing compounds, whereas STL may contain tea phenolics, catechin‐derived compounds, minerals, and processing‐related transformation products (Prawira‐Atmaja and Puangpraphant 2025; Basso et al. 2025; Choe 2025; Çakmak et al. 2024). Such differences may influence fungal metabolism and nutrient uptake during cultivation, leading to distinct compositional outcomes in the fruiting bodies. This suggests that these residues may function not only as alternative substrate components, but also as formulation inputs for shaping the nutritional and biochemical profile of edible mushrooms.

*Hypsizygus ulmarius* is suitable for this type of investigation because it combines edible value, reported bioactive properties, and practical cultivation advantages. It is appreciated as an edible mushroom and has attracted attention due to its potential health‐promoting properties (Thimmaraju and Seedevi 2024; Bhatia and Yadav 2025). In addition, *H. ulmarius* can be cultivated relatively easily compared with several other mushroom species, which increases its relevance for both commercial and small‐scale production systems (Atila 2023). These characteristics make it a useful model for examining whether alternative substrate inputs can modulate the nutritional and compositional traits of harvested fruiting bodies. In this context, the search for alternative substrate inputs is relevant not only for modifying fruiting body composition but also for incorporating underused organic residues into value‐added mushroom production systems. Spent tea leaves and spent coffee grounds are suitable candidates for this purpose because they are abundantly generated after beverage preparation and contain residual organic and bioactive constituents that may influence the nutritional and antioxidant characteristics of the harvested mushrooms.

Previous studies have reported that, in *H. ulmarius*, substrate‐related differences can be reflected in antioxidant capacity, amino acid and fatty acid composition, vitamin and mineral contents, as well as in structural or metabolite‐related features evaluated using techniques such as FTIR, SEM, particle size analysis, and GC–MS profiling (Atíla 2022; Hausiku and Mupambwa 2018; Chauhan et al. 2024; Hausiku‐Ikechukwu et al. 2024; Aditya et al. 2025; Nwaru et al. 2026). FTIR analysis is particularly useful as a complementary tool because it provides spectral information related to major functional groups associated with proteins, polysaccharides, lipids, and phenolic‐related compounds in the fungal matrix (Baltacıoğlu et al. 2017; Bekiaris et al. 2020). Nevertheless, direct comparisons of chemically distinct post‐brewing residues under identical cultivation conditions and graded inclusion levels remain limited. In particular, it is still unclear whether STL and SCG induce distinct and coordinated changes across multiple compositional domains within the same mushroom system, or whether their effects are restricted to a few isolated traits.

Accordingly, this study evaluated whether spent tea leaves and spent coffee grounds produce distinct compositional responses in *H. ulmarius* fruiting bodies cultivated on wheat straw‐based substrates under identical conditions. We hypothesized that the contrasting chemical characteristics of STL and SCG would result in residue‐specific changes in nutritional composition, antioxidant‐associated properties, and FTIR spectral features, rather than a simple dose‐dependent enrichment pattern. To test this hypothesis, fruiting bodies were analyzed for proximate composition, macro‐ and micro‐elements, amino acid and fatty acid profiles, phenolic‐related traits, antioxidant activity, and FTIR spectra. The resulting dataset was used to assess whether post‐brewing residues can serve as practical substrate formulation inputs.

## 2 Material and Methods

### 2.1 Chemicals and Reagents

All solvents and reagents were of analytical or chromatographic grade. Standards and reference compounds used for amino acid, fatty acid, phenolic‐related, antioxidant, and spectroscopic analyses were obtained from Sigma‐Aldrich (St. Louis, MO, USA). Ultrapure water was used throughout all analytical procedures.

### 2.2 Mushroom Strain, Substrate Preparation, and Cultivation

The *H. ulmarius* strain HU‐NL was obtained from the Mushroom Research and Application Center of Kırşehir Ahi Evran University. Stock cultures were maintained on potato dextrose agar (PDA) at 4°C.

Wheat straw (WS) was used as the basal substrate. Spent tea leaves (STL) and spent coffee grounds (SCG) were collected from a local branch of an international coffee chain after beverage preparation. The residues were transported to the laboratory on the same day, manually inspected to remove visible foreign materials, and dried at 40°C to constant weight to reduce moisture and improve storage stability. The dried materials were stored in sealed polyethylene bags at room temperature until use. The initial chemical characteristics of STL and SCG, including proximate composition, phenolic‐related traits, antioxidant activity, and selected mineral elements, are provided in Table S1. The same analytical procedures described below for fruiting bodies were also applied to STL and SCG for proximate composition, phenolic‐related traits, antioxidant activity, and mineral analysis.

Prior to substrate preparation, dried STL and SCG were manually homogenized and incorporated into wheat straw at 10%, 20%, and 30% (w/w), yielding WS90:STL10, WS80:STL20, WS70:STL30, WS90:SCG10, WS80:SCG20, and WS70:SCG30 formulations; wheat straw alone served as the control. Substrate moisture content was adjusted to 65%. The substrates were packed into polypropylene bags (1 kg wet weight per bag), sterilized in an autoclave at 121°C under 1 atm pressure for 90 min, cooled to room temperature, and inoculated with 3% spawn (w/w).

Incubation was carried out at 25°C in the dark until full mycelial colonization. Fruiting was induced at 18°C and 80%–90% relative humidity under fluorescent light (200–250 lx). Fruiting bodies from the first flush were harvested at commercial maturity, defined as the stage at which the in‐rolled margins of the pileus began to flatten (Öztürk and Atila 2021), and used for all compositional analyses.

Each treatment consisted of 10 cultivation bags. The experiment was conducted according to a completely randomized design (CRD), and cultivation bags were randomly arranged within the incubation and fruiting rooms to minimize positional effects.

### 2.3 Sample Collection and Preparation

Mushrooms from the control and six supplemented formulations were collected as three independent biological replicates per treatment. After harvest, fruiting bodies were cleaned of visible substrate residues, cut into small pieces, dried at 40°C to constant weight, and ground to a fine powder. The powdered samples were stored at 4°C until analysis. All analytical measurements were performed in triplicate, and results are expressed as mean ± standard deviation.

### 2.4 Proximate Composition

Moisture, total protein, crude fat, and ash contents were determined according to AOAC International procedures (AOAC 2021). Moisture content was measured gravimetrically by oven drying at 105°C (Nuve, FN 400P) to constant weight. Total protein was determined by the Kjeldahl method and calculated from total nitrogen using a conversion factor of 4.38. Crude fat was measured by Soxhlet extraction with hexane, and ash content was determined by incineration in a muffle furnace at 550°C for 4–6 h until white or gray ash was obtained. Total carbohydrate content was determined analytically by the phenol–sulfuric acid method using glucose as the calibration standard (Dubois et al. 1956). Accordingly, the carbohydrate values reported in this study represent analytically determined total carbohydrate content rather than carbohydrate estimated by difference.

### 2.5 Amino Acid Analysis

Total amino acid composition was determined by HPLC after hydrolysis, using the Agilent amino acid analysis protocol with minor modifications (Agilent Technologies 2025). All amino acids except tryptophan were determined after 6 N HCl hydrolysis, whereas tryptophan was determined separately after alkaline hydrolysis following an AOAC‐compatible approach. For acid hydrolysis, dried mushroom powder (0.5 g) was treated with 5 mL of 6 N HCl in sealed glass vials at 110°C for 24 h. To reduce oxidative degradation and improve the recovery of hydrolysis‐sensitive amino acids, 250 μL of 2 mM phenol and 2 mL of 2% 3,3′‐dithiodipropionic acid (DTDPA) were added prior to hydrolysis.

After hydrolysis, samples were neutralized to pH 6.7–7.3, brought to 100 mL with ultrapure water, centrifuged at 4000 rpm for 5 min, and filtered. The hydrolysates were then subjected to pre‐column derivatization according to the manufacturer's protocol for the Eclipse AAA method. Chromatographic analyses were performed on an Agilent 1260 Infinity II HPLC system equipped with a diode array detector set at 338 nm and an Agilent Eclipse AAA column (5 μm, 150 × 4.6 mm). The mobile phases consisted of 40 mM borate buffer (pH 7.8) and acetonitrile:methanol:water (45:45:10, v/v/v), and the gradient program is given in Table S2. Amino acids were identified and quantified by comparison with the retention times and peak areas of a 17‐amino‐acid standard mixture (Sigma‐Aldrich, USA). Results were expressed as mg/100 g dry weight. Tryptophan was determined separately by alkaline hydrolysis (4 M NaOH, 110°C, 16 h), since it is degraded under standard acid hydrolysis conditions (Bech‐Andersen 1991).

### 2.6 Chemical Amino Acid Score Calculation

Chemical amino acid scores were calculated to provide a preliminary evaluation of the amino acid adequacy of the protein fraction. Amino acid contents determined on a dry‐weight basis (mg/100 g DW) were first converted to mg amino acid per g protein using the protein contents obtained from proximate composition analysis:

$$\begin{array}{c} \mathrm{mg}\;\text{amino acid}/g\;\text{protein}=\text{amino acid content}\\ \left(\mathrm{mg}/100\;g\;\mathrm{DW}\right)/\text{protein content}\;\left(g/100\;g\;\mathrm{DW}\right) \end{array}$$

The calculated amino acid values were compared with the FAO/WHO/UNU adult indispensable amino acid scoring pattern (FAO/WHO/UNU 2007), expressed as mg/g protein. Chemical amino acid score was calculated as follows:

$$\begin{array}{c} \text{Chemical score}\;\left(\%\right)=[\text{sample amino acid or amino acid group}\\ \left(\mathrm{mg}/g\;\text{protein}\right)/\text{reference amino acid pattern}\;\left(\mathrm{mg}/g\;\text{protein}\right)]\times 100 \end{array}$$

For grouped amino acids, sulfur‐containing amino acids were calculated as methionine+cystine, and aromatic amino acids as phenylalanine+tyrosine. The amino acid or amino acid group with the lowest score was considered the limiting amino acid. Because digestibility was not determined in the present study, chemical scores were interpreted only as preliminary compositional indicators and were not used to calculate digestibility‐corrected protein quality indices such as the protein digestibility‐corrected amino acid score (PDCAAS) or the digestible indispensable amino acid score (DIAAS).

### 2.7 Fatty Acid Composition

Fatty acid composition was determined by gas chromatography after conversion of extracted lipids to fatty acid methyl esters (FAMEs). Lipid extracts obtained during Soxhlet analysis were used for methyl ester preparation following the official IOC COI/T.20/Doc. No. 33 method (International Olive Council 2024), adapted for mushroom‐derived lipids. Briefly, an aliquot of the recovered lipid extract was transesterified with methanolic KOH prior to GC analysis. FAMEs were separated on an Agilent 6890 GC‐FID system fitted with an HP‐88 capillary column (100 m × 0.25 mm i.d., 0.20 μm film thickness). Injector and detector temperatures were maintained at 250°C and 260°C, respectively, with a split ratio of 1:100. The oven program consisted of an initial hold at 120°C for 1 min, heating to 240°C at 4°C/min, and a final hold at 240°C for 5 min. Fatty acids were identified by comparison of retention times with those of a 37‐component FAME standard mixture (Supelco, Bellefonte, PA, USA). Results were expressed as the percentage of total identified fatty acids based on relative peak areas.

### 2.8 Elemental Analysis

For elemental analysis, 0.25 g of dried mushroom powder was digested in a Berghof‐MWS2 microwave digestion system (Berghof speedwave, Germany) using 9 mL of 65% HNO_3_ and 1 mL of 30% H_2_O_2_. After digestion, the final volume was adjusted to 50 mL with deionized water, and reagent blanks were prepared in parallel. The digestion program involved heating to 180°C within 5 min, holding for 2 min, and repeating the cycle once more (Sarikurkcu et al. 2011). Concentrations of P, K, Ca, Mg, Na, Fe, Mn, Zn, and Cu were determined by ICP‐MS (Thermo Scientific XSeries II) and expressed as mg/kg dry weight. Quantification was based on multi‐element external calibration, and instrument stability was checked using continuing calibration verification (CCV) solutions. Accuracy and precision were assessed using SRM 1547 as a certified reference material. Certified values, measured concentrations, recoveries (%), and coefficients of variation (CV, %) are presented in Table S3. Additional analytical performance characteristics are provided in the Supporting Information.

### 2.9 Phenolic‐Related Traits and Antioxidant Activity

Methanolic extracts were prepared according to the methods described by Sezer et al. (2017) and Lu et al. (2014), with some modifications. Briefly, 1 g of dried mushroom powder was mixed with 10 mL of 80% methanol and subjected to ultrasonic‐assisted extraction for 30 min in the dark. As an additional extraction step, the samples were incubated at 60°C for 16 h in a shaking water bath. After centrifugation at 5000 rpm for 15 min, the supernatants were collected for analysis.

Total phenolic content (TPC) was determined by the Folin–Ciocalteu method (Singleton and Rossi 1965). Absorbance was measured at 765 nm, and quantification was performed using a gallic acid calibration curve. Results were expressed as mg gallic acid equivalents (GAE)/g DW. Total flavonoid content (TFC) was measured by the AlCl_3_ colorimetric method (Meda et al. 2005). Absorbance was recorded at 415 nm using quercetin as the standard, and results were expressed as mg quercetin equivalents (QE)/g DW. Total monomeric anthocyanin content (TAM) was determined by the pH differential method (Lee et al. 2005) and expressed as mg cyanidin‐3‐glucoside equivalent/L extract. Because anthocyanins are not considered major natural pigments in mushrooms, TAM was included only as an exploratory anthocyanin‐equivalent phenolic‐related index and was not interpreted as evidence of major anthocyanin‐type pigmentation in *H. ulmarius*.

Antioxidant capacity was evaluated using ABTS radical cation scavenging activity (Hasbal et al. 2015), DPPH radical scavenging activity (Xu and Chang 2007), and CUPRAC (Apak et al. 2007). Absorbance was measured at 734 nm for ABTS, 517 nm for DPPH, and 450 nm for CUPRAC. ABTS values were expressed as μmol Trolox equivalents (TE)/g DW, whereas DPPH and CUPRAC values were expressed as μmol ascorbic acid equivalents (AAE)/g DW.

### 2.10 FTIR Spectroscopy

FTIR spectra of powdered mushroom samples were recorded using an IRTracer‐100 spectrometer (Shimadzu, Japan) equipped with an ATR accessory and a DLATGS detector. Spectra were collected over the 4000–400 cm^−1^ range at a resolution of 4 cm^−1^ with 64 accumulated scans per sample, following D'Souza and Kamat (2017). Spectral preprocessing was performed using the instrument software. Spectra were baseline‐corrected before comparative evaluation of band positions and relative spectral profiles. FTIR was used as a complementary qualitative molecular characterization tool to assess major functional groups associated with polysaccharides, protein/chitin‐related structures, lipids, and other matrix components. Band assignments were interpreted with reference to the relevant literature.

### 2.11 Statistical Analysis

Ten cultivation bags were prepared for each treatment to obtain sufficient fruiting body material under the same cultivation conditions. For compositional analyses, three bags were randomly selected from the ten cultivation bags of each treatment. Fruiting bodies harvested from each selected bag were processed separately, and each selected bag was considered an independent biological replicate. Thus, the chemical, nutritional, antioxidant, and FTIR analyses were performed using three independent biological replicates per treatment. Analytical measurements were performed in triplicate, and analytical replicate values were averaged before statistical evaluation. Biological replicates were used as the experimental units. Results are presented as mean ± standard deviation (SD). Statistical comparisons among treatments were performed by one‐way analysis of variance (ANOVA), followed by Tukey's multiple comparison test at *p* < 0.05. Before ANOVA, data were checked for normality and homogeneity of variance using the Shapiro–Wilk and Levene tests, respectively.

Principal component analysis (PCA) was performed to evaluate integrated variation among treatments using proximate composition, amino acid, fatty acid, mineral, phenolic‐related, and antioxidant variables. PCA was conducted using treatment‐level mean values to visualize overall compositional patterns. Prior to analysis, all variables were autoscaled (mean‐centered and scaled to unit variance) to eliminate unit‐related bias. FTIR data were not included in the multivariate statistical analysis because they were interpreted qualitatively as supportive structural information. All statistical analyses were carried out using SPSS version 16.0 (IBM Corp., Armonk, NY, USA).

## 3 Results and Discussion

### 3.1 Proximate Composition

Substrate formulation affected all proximate composition variables of *H. ulmarius* fruiting bodies (*p* < 0.001; Table 1). Except for moisture, values are expressed on a dry‐weight basis. Moisture ranged from 89.94% to 93.30%, ash from 7.67 to 8.87 g/100 g, protein from 14.63 to 17.37 g/100 g, crude lipid from 2.00 to 2.50 g/100 g, and total carbohydrate from 39.7 to 45.6 g/100 g. Overall, supplementation altered the relative distribution of major proximate fractions rather than producing uniform increases across all components.

**TABLE 1:** Nutritional composition of mushrooms cultivated under different growing media.

Substrates	Moisture (%)	Ash (g/100 g)	Protein (g/100 g)	Crude lipid (g/100 g)	Carbonhydrate (g/100 g)
Control	89.94 ± 0.48 d	7.67 ± 0.33 c	14.63 ± 0.12 e	2.23 ± 0.06 b	45.6 ± 0.3 a
WS90: STL10	93.30 ± 0.78 a	8.40 ± 0.16 ab	15.53 ± 0.12 cd	2.07 ± 0.03 c	42.3 ± 0.1 b
WS80: STL20	91.20 ± 0.24 cd	8.63 ± 0.12 a	16.77 ± 0.24 b	2.00 ± 0.07 c	39.7 ± 0.2 c
WS70:STL30	92.87 ± 0.25 ab	8.87 ± 0.12 a	17.37 ± 0.12 a	2.10 ± 0.04 c	41.2 ± 0.4 bc
WS90: SCG10	91.03 ± 0.39 cd	7.93 ± 0.05 bc	15.20 ± 0.14 d	2.40 ± 0.02 a	44.3 ± 0.7 a
WS80: SCG20	91.70 ± 0.22 bc	7.97 ± 0.12 bc	15.63 ± 0.06 c	2.41 ± 0.03 a	44.6 ± 0.6 a
WS70:SCG30	92.57 ± 0.34 ab	7.83 ± 0.21 bc	15.73 ± 0.12 c	2.50 ± 0.01 a	39.8 ± 0.6 c
*p* value	< 0.001	< 0.001	< 0.001	< 0.001	< 0.001

*Note:* Each value is expressed as mean ± standard deviation (*n* = 3). Values within the same column sharing the same letter are not significantly different according to Tukey's HSD test.

STL and SCG produced different compositional responses. In the STL series, ash and protein showed the most evident increases. Ash increased from 7.67 g/100 g in the control to the highest value of 8.87 g/100 g in WS70:STL30, while protein increased from 14.63 to 17.37 g/100 g in the same treatment (Table 1), representing an increase of approximately 18.7% in protein content. Carbohydrate content was lower than the control in the STL treatments, indicating a shift toward a protein‐enriched proximate profile. In contrast, crude lipid content in STL treatments remained below the control value (2.23 g/100 g), ranging from 2.00 to 2.10 g/100 g, without a clear increasing trend.

SCG supplementation showed a different pattern. Protein increased only moderately, reaching 15.73 g/100 g in WS70:SCG30, whereas crude lipid content increased from 2.23 g/100 g in the control to 2.40–2.50 g/100 g in SCG‐supplemented treatments. Carbohydrate content was lowest in WS70:SCG30, although lower SCG levels remained close to the control. Thus, SCG was more closely associated with lipid‐related changes, whereas STL was more strongly linked to protein‐ and ash‐related shifts.

These findings indicate that STL and SCG generated distinct formulation‐dependent proximate profiles in *H. ulmarius* rather than acting as interchangeable supplementation materials. Similar substrate‐related changes in proximate composition have been reported in edible mushrooms grown on agro‐industrial residues (Sardar et al. 2017; Atíla 2022; Jasinska et al. 2024; Chen et al. 2025; Chauhan et al. 2024; Alsanad et al. 2021). The present results extend these observations by showing that two chemically different post‐brewing residues, applied under the same cultivation conditions, redirected different proximate fractions instead of producing a general enrichment response. Because the chemical composition of STL and SCG was characterized (Table S1), the divergent responses observed in *H. ulmarius* can be linked to differences in nitrogenous compounds, residual lipids, mineral profiles, soluble carbohydrates, and extractable bioactive constituents, which are likely to influence nutrient assimilation and compositional partitioning during fruiting body development. Therefore, these responses should be interpreted as residue‐associated compositional patterns rather than direct mechanistic evidence of substrate‐to‐fruiting‐body transfer.

### 3.2 Macro‐ and Micro‐Element Composition

Mineral composition differed among substrate formulations (*p* < 0.001; Tables 2 and 3), with pairwise treatment differences indicated by the lowercase letters in the tables. Across treatments, K was the predominant macro‐element, followed by P, Mg, Na, and Ca. Although this hierarchy was generally maintained, STL and SCG produced element‐specific differences in mineral concentration patterns.

**TABLE 2:** Macroelement contents of mushrooms cultivated under different growing media.

Growing media	P (mg/kg)	K (mg/kg)	Ca (mg/kg)	Mg (mg/kg)	Na (mg/kg)
Control	6954.0 ± 57.6 f	30457.4 ± 926.2 c	72.9 ± 1.3 e	1605.6 ± 5.5 c	872.2 ± 11.7 d
WS90: STL10	6964.7 ± 49.9 f	28835.7 ± 743.5 d	89.0 ± 0.8 b	1673.6 ± 6.0 b	1140.6 ± 45.5 b
WS80: STL20	7940.2 ± 50.5 d	30411.8 ± 540.2 c	68.2 ± 1.0 f	1686.6 ± 8.7 b	926.3 ± 18.5 c
WS70:STL30	7379.0 ± 129.3 e	25594.8 ± 939.2 e	93.0 ± 1.9 a	1438.7 ± 12.9 e	864.5 ± 9.4 d
WS90: SCG10	9756.0 ± 62.1 c	35030.7 ± 657.5 a	81.5 ± 1.3 c	1827.2 ± 29.4 a	1243.3 ± 36.4 a
WS80: SCG20	10151.6 ± 125.3 b	28152.5 ± 362.7 d	73.6 ± 0.8 e	1516.4 ± 15.2 d	711.0 ± 8.4 f
WS70:SCG30	12864.1 ± 373.9 a	32318.3 ± 457.1 b	80.8 ± 2.3 d	1835.6 ± 27.8 a	767.4 ± 24.4 e
*p* value	< 0.001	< 0.001	< 0.001	< 0.001	< 0.001

*Note:* Each value is expressed as mean ± standard deviation (*n* = 3). Values within the same column sharing the same letter are not significantly different according to Tukey's HSD test.

**TABLE 3:** Micro‐element contents of mushrooms cultivated under different growing media.

Growing media	Fe (mg/kg)	Zn (mg/kg)	Mn (mg/kg)	Cu (mg/kg)
Control	61.2 ± 1.9 e	52.2 ± 0.3 c	7.7 ± 0.1 e	12.1 ± 0.2 bc
WS90: STL10	63.0 ± 2.0 e	44.0 ± 1.3 e	8.4 ± 0.2 d	8.6 ± 0.2 e
WS80: STL20	73.6 ± 3.0 c	51.1 ± 1.0 c	9.3 ± 0.2 b	9.6 ± 0.3 d
WS70:STL30	67.3 ± 0.5 d	46.1 ± 1.6 d	10.0 ± 0.1 a	8.7 ± 0.1 e
WS90: SCG10	74.8 ± 0.6 c	57.6 ± 2.1 b	8.9 ± 0.1 c	12.4 ± 0.4 c
WS80: SCG20	106.0 ± 4.9 a	53.6 ± 1.1 c	9.4 ± 0.2 b	11.9 ± 0.8 b
WS70:SCG30	91.9 ± 2.0 b	61.6 ± 2.1 a	10.3 ± 0.6 a	15.0 ± 0.1 a
*p* value	< 0.001	< 0.001	< 0.001	< 0.001

*Note:* Each value is expressed as mean ± standard deviation (*n* = 3). Values within the same column sharing the same letter are not significantly different according to Tukey's HSD test.

The most pronounced macro‐element responses were observed in SCG‐supplemented treatments. P reached its maximum in WS70:SCG30 (12,864.1 mg/kg), nearly 1.85‐fold higher than the control, while Mg also peaked in WS70:SCG30 (1835.6 mg/kg). The highest K concentration was observed in WS90:SCG10 (35,030.7 mg/kg). In contrast, Ca reached its maximum in WS70:STL30 (93.0 mg/kg), suggesting a more specific association between STL supplementation and Ca concentration. Na differed among treatments but did not follow a clear dose‐dependent pattern.

Micro‐element composition also varied with formulation. Within the SCG series, Fe Fe reached its highest concentration in WS80:SCG20 (106.0 mg/kg), whereas Zn and Cu reached their highest values in WS70:SCG30, with 61.6 and 15.0 mg/kg, respectively (Table 3). Mn also increased modestly across supplemented treatments. Overall, SCG was associated with a broader increase in trace‐element concentrations than STL, particularly for Fe, Zn, and Cu.

These results indicate that STL and SCG shaped mineral concentration patterns in *H. ulmarius* in different ways. This residue‐specific response is consistent with previous studies showing that mineral accumulation in mushroom fruiting bodies depends strongly on both species and substrate formulation. Lee et al. (2009), for example, examined mineral transfer from cultivation substrates to the fruiting bodies of *Pleurotus eryngii*, *Flammulina velutipes*, and *Hypsizygus marmoreus*, in which K, Mg, Ca, and Na were the dominant mineral elements in both substrates and mushrooms. In a broader comparison, Siwulski et al. (2019) studied six cultivated species, including *Agrocybe cylindracea*, *Clitocybe maxima*, *Flammulina velutipes*, *Ganoderma lucidum*, *Lentinula edodes*, and *Pleurotus eryngii*, grown on two sawdust‐based substrate systems differing in nutritional additives, and demonstrated that substrate composition can alter macro‐ and trace‐element accumulation. Residue‐based effects have also been reported for *Pleurotus ostreatus* cultivated with tea waste, where the waste‐containing substrate modified the nutritional composition of the fruiting bodies (Ahmed et al. 2024). In this context, the present study adds evidence that chemically distinct post‐brewing residues do not produce a uniform mineral enrichment pattern; rather, SCG was more closely associated with P, Mg, K, Fe, Zn, and Cu, whereas STL showed a narrower association with Ca.

### 3.3 Phenolic‐Related Traits and Antioxidant Activity

Phenolic‐related traits and antioxidant activity were evaluated as composition‐related quality indicators because they reflect the extractable redox‐active fraction of the fruiting bodies and provide information on radical‐scavenging and reducing‐capacity responses. These parameters do not directly describe sensory quality, but they contribute to the compositional characterization of mushrooms as food materials. Phenolic‐related traits and antioxidant properties differed among formulations (*p* < 0.001; Table 4). The responses varied according to residue type and assay, indicating that STL and SCG did not produce a single antioxidant pattern.

**TABLE 4:** Total phenolic content and flavonoid content, antioxidant activity (ABTS, CUPRAC, DPPH), and total monomeric anthocyanin levels of mushrooms cultivated on different substrate formulations.

Substrates	Total phenol (mg GAE/g)	Total flavonoid (mg QE/g)	ABTS (μmol TE/g dw)	CUPRAC (μM AAS/g dw)	DPPH (mM AAE/g)	Total monomeric anthocyanin (mg/L)
Control	4.92 ± 0.14 a	0.70 ± 0.007 a	11.19 ± 0.102 c	13.74 ± 0.35 c	1.97 ± 0.07 a	0.74 ± 0 a
WS90: STL10	5.01 ± 0.09 a	0.32 ± 0.002 d	9.43 ± 0.076 d	14.38 ± 0.03 c	0.69 ± 0.02 c	0.65 ± 0.002 c
WS80: STL20	2.49 ± 0.07 e	0.11 ± 0.005 g	8.04 ± 0.007 e	16.21 ± 0.098 b	0.09 ± 0.02 e	0.42 ± 0.003 e
WS70:STL30	2.51 ± 0.06 e	0.29 ± 0.008 e	11.94 ± 0.045 b	17.76 ± 0.6 a	0.35 ± 0.03 d	0.56 ± 0.02 d
WS90: SCG10	3.41 ± 0.02 c	0.60 ± 0.002 b	13.94 ± 0.12 a	15.7 ± 0.64 c	1.12 ± 0.08 b	0.69 ± 0 b
WS80: SCG20	4.63 ± 0.06 b	0.49 ± 0.01 c	14.09 ± 0.11 a	13.5 ± 0.069 c	1.22 ± 0.04 b	0.69 ± 0.01 b
WS70:SCG30	3.00 ± 0.05 d	0.24 ± 0.001 f	12.28 ± 0.34 b	14.16 ± 0.4 c	0.64 ± 0.02 c	0.57 ± 0.01 d
*p* value	< 0.001	< 0.001	< 0.001	< 0.001	< 0.001	< 0.001

*Note:* Each value is expressed as mean ± standard deviation (*n* = 3). Values within the same column sharing the same letter are not significantly different according to Tukey's HSD test. All analyses were performed on alcoholic extracts using spectrophotometric methods.

Abbreviations: AAE, ascorbic acid equivalent; ABTS, 2,2′‐azinobis‐(3‐ethylbenzothiazoline‐6‐sulfonic acid); CUPRAC, cupric ion reducing antioxidant capacity; DPPH, 2,2‐diphenyl‐1‐picrylhydrazyl; dw, dry weight; GAE, gallic acid equivalent; QE, quercetin equivalent; TE, Trolox equivalent.

Among phenolic‐related traits, the highest TPC values were observed in WS90:STL10 (5.01 mg GAE/g) and the control (4.92 mg GAE/g). Higher STL levels reduced TPC markedly, whereas within the SCG series, TPC was highest in WS80:SCG20 (4.63 mg GAE/g). TFC declined in all supplemented treatments relative to the control, with the strongest decreases observed in the STL series. Because AlCl3‐based measurements in mushrooms do not necessarily reflect classical plant‐type flavonoids, TFC values should be interpreted as a flavonoid‐equivalent index rather than direct evidence of flavonoid accumulation (Gil‐Ramírez et al. 2016). Overall, SCG, particularly at 20%, maintained higher extractable phenolic‐related indices than most other supplemented treatments, whereas higher STL levels were associated with greater declines.

Antioxidant assays showed non‐parallel responses. ABTS values were highest in SCG‐supplemented treatments, particularly WS80:SCG20, whereas WS80:STL20 showed the lowest response. DPPH followed a broadly similar trend, with WS80:SCG20 giving the highest value among supplemented treatments, while STL treatments remained lower. In contrast, CUPRAC increased across the STL series and reached its maximum in WS70:STL30 (17.76 μM AAE/g). ABTS and DPPH mainly reflect radical‐scavenging activity, whereas CUPRAC reflects the reducing capacity of electron‐donating compounds (Apak et al. 2007). Therefore, SCG was more closely associated with radical‐scavenging responses, whereas high STL inclusion was associated with greater reducing capacity.

TAM values were detected at low levels across all treatments and varied within a narrow range (0.42–0.74 mg/L). The control showed the highest TAM value, while among supplemented treatments the highest values were observed in WS90:SCG10 and WS80:SCG20. STL supplementation, particularly at the intermediate level, was associated with lower TAM values. Because anthocyanins are not considered major natural pigments in mushrooms and the overall TAM range was limited, TAM should be regarded only as a secondary anthocyanin‐equivalent compositional indicator rather than evidence of major anthocyanin‐type pigmentation or a major determinant of antioxidant behavior in this system. The antioxidant behavior of *H. ulmarius* could not be explained by TPC, TFC, or TAM alone. Similar substrate‐dependent variation in phenolic‐related traits has been reported in cultivated mushrooms grown on agro‐industrial residues (Atíla 2022; Koutrotsios et al. 2022; Diamantopoulou et al. 2023), but the present results refine this interpretation by showing that residue supplementation does not necessarily increase phenolic‐related indices. Instead, residue type and inclusion level shaped assay‐specific redox responses. The non‐parallel behavior among TPC, TFC, TAM, and antioxidant assays may reflect the contribution of non‐phenolic reducing compounds, matrix‐dependent extractability, Maillard‐type products, residual coffee‐ or tea‐derived compounds, or assay‐specific reaction mechanisms. These in vitro assays describe extractable redox‐related properties and should not be interpreted as direct evidence of physiological antioxidant effects. Nevertheless, they show that post‐brewing residues can differentially influence redox‐related compositional traits rather than producing a uniform antioxidant response.

### 3.4 Amino Acid Composition

Amino acid composition differed among substrate formulations (*p* < 0.001; Table 5), with pairwise treatment differences indicated by the lowercase letters. On a dry‐weight basis, total essential amino acids ranged from 673.0 to 970.5 mg/100 g, and total non‐essential amino acids ranged from 2469.8 to 3259.7 mg/100 g. The highest total amino acid content was recorded in WS70:STL30 (4230 mg/100 g), representing an approximately 32% increase in both essential and non‐essential amino acid fractions relative to the control. This treatment also showed the highest protein content (Table 1), indicating consistency between crude protein and amino acid profiles.

**TABLE 5:** Amino acid composition of *Hypsizygus ulmarius cul*tivated under different growing medium formulations.

Amino acids (mg/100 g)			Control	WS90:STL10	WS80:STL20	WS70:STL30	WS90:SCG10	WS80:SCG20	WS70:SCG30	*p* value
Essential amino acids	Valine	Val	128.67 ± 2.87 a	127.33 ± 2.62 ab	121.00 ± 2.45 b	129.67 ± 1.30 a	113.33 ± 2.87 c	105.33 ± 1.30 d	121.00 ± 1.24 b	< 0.001
	Isoleucine	Ile	65.33 ± 1.25 c	66.00 ± 0.82 c	69.00 ± 0.79 c	82.83 ± 1.03 b	67.67 ± 1.25 c	83.33 ± 1.27 b	87.67 ± 2.10 a	< 0.001
	Leucine	Leu	240.67 ± 2.90 c	221.33 ± 2.10 e	235.33 ± 1.70 cd	271.67 ± 4.92 a	230.00 ± 2.16 d	265.00 ± 3.27 ab	260.33 ± 2.85 b	< 0.001
	Threonine	Thr	73.67 ± 0.94 de	77.67 ± 2.05 cd	91.67 ± 3.30 b	116.33 ± 2.87 a	68.00 ± 0.80 e	68.33 ± 0.47 e	82.33 ± 1.23 c	< 0.001
	Methionine	Met	22.33 ± 1.30 c	22.43 ± 0.50 c	23.67 ± 0.47 c	38.33 ± 0.47 a	16.33 ± 0.94 d	22.67 ± 0.45 c	34.33 ± 0.50 b	< 0.001
	Phenylalanine	Phe	96.33 ± 126 c	94.67 ± 1.70 c	104.33 ± 1.72 b	113.33 ± 1.25 a	80.33 ± 1.24 d	97.00 ± 2.15 c	96.67 ± 0.48 c	< 0.001
	Lysine	Lys	66.67 ± 2.05 e	27.00 ± 0.82 g	41.67 ± 0.43 f	174.67 ± 6.94 a	130.67 ± 2.62 c	82.33 ± 1.26 d	143.33 ± 3.68 b	< 0.001
	Histidine	His	28.00 ± 0.80 a	24.67 ± 0.47 c	24.00 ± 0.85 c	27.00 ± 0.90 b	26.00 ± 0.84 bc	20.33 ± 0.45 d	19.67 ± 0.47 d	< 0.001
	Trptophan	Trp	11.77 ± 0.56 b	12.00 ± 0.80 b	11.67 ± 0.47 b	16.67 ± 0.46 a	18.00 ± 0.41 a	11.67 ± 0.49 b	18.33 ± 0.94 a	< 0.001
	Total		733.43	673.00	722.33	970.50	750.33	756.00	863.67	
	Arginine	Arg	660.67 ± 2.05 g	726.00 ± 3.74 e	817.00 ± 5.35 c	969.00 ± 12.90 a	700.00 ± 4.55 f	778.33 ± 9.74 d	850.33 ± 11.44 b	< 0.001
	Aspartic acid	Asp	90.00 ± 0.82 d	92.33 ± 0.94 c	95.67 ± 2.05 c	85.33 ± 1.24 cd	106.67 ± 2.36 b	101.67 ± 2.08 b	111.33 ± 3.09 a	< 0.001
	Glutamic acid	Glu	258.67 ± 3.30 d	263.67 ± 2.87 d	331.33 ± 2.10 a	339.33 ± 1.70 a	295.67 ± 4.78 c	306.33 ± 2.87 b	295.00 ± 5.72 c	< 0.001
	Alanine	Ala	377.00 ± 4.55 d	392.67 ± 4.64 c	371.00 ± 4.90 d	417.67 ± 4.11 b	365.33 ± 2.07 d	242.67 ± 1.28 e	439.67 ± 4.78 a	< 0.001
	Glycine	Gly	465.00 ± 7.48 f	659.00 ± 4.97 e	788.00 ± 5.35 cd	843.00 ± 18.18 b	771.33 ± 5.44 d	805.33 ± 4.99 c	877.33 ± 9.46 a	< 0.001
	Tyrosine	Tyr	264.67 ± 6.94 a	87.67 ± 1.25 d	195.00 ± 3.74 b	155.67 ± 2.49 c	51.67 ± 0.94 e	55.33 ± 0.48 e	54.33 ± 0.94 e	< 0.001
Non‐essential amino acids	Asparagine	Asn	16.50 ± 0.41 bc	14.97 ± 0.42 d	15.67 ± 0.25 cd	17.73 ± 0.38 a	15.63 ± 0.39 cd	17.10 ± 0.14 ab	15.83 ± 0.24 cd	< 0.001
	Glutamine	Gln	67.33 ± 1.26 bc	64.33 ± 0.47 c	70.33 ± 1.30 b	75.33 ± 1.26 a	66.33 ± 1.23 c	56.67 ± 0.29 d	66.33 ± 1.70 c	< 0.001
	Cystine	Cys	138.67 ± 2.60 e	148.00 ± 2.94 d	131.67 ± 1.28 e	200.00 ± 3.74 a	164.00 ± 3.56 c	191.00 ± 3.27 b	196.67 ± 2.49 ab	< 0.001
	Norvaline	Nva	45.00 ± 0.81 e	46.67 ± 0.48 e	44.67 ± 0.45 e	51.67 ± 0.46 d	62.67 ± 1.28 c	73.00 ± 1.63 b	87.33 ± 2.05 a	< 0.001
	Hdroxyproline	Hyp	52.00 ± 0.82 c	52.33 ± 1.26 c	111.00 ± 1.63 a	74.67 ± 1.71 b	33.33 ± 0.48 e	40.00 ± 0.80 d	39.33 ± 0.47 d	< 0.001
	Proline	Pro	34.33 ± 0.47 d	35.67 ± 0.45 d	36.33 ± 0.94 d	30.33 ± 1.25 e	54.33 ± 1.26 c	89.67 ± 2.07 a	77.00 ± 0.82 b	< 0.001
	Total		2469.83	2583.00	3007.67	3259.67	2686.67	2757.67	3010.17	

*Note:* Value (mg/100 g) = mean ± SD (*n* = 3). Different lowercase letters within the same row indicate significant differences among treatments according to Tukey’s HSD test (*P* < 0.05).

Abbreviation: ND, not detectable.

The essential amino acid fraction showed a residue‐dependent response, with the broadest increase observed in the STL series. WS70:STL30 produced the highest total essential amino acid content and exhibited elevated levels for several individual essential amino acids, whereas SCG treatments induced more selective changes. For example, WS70:SCG30 showed the highest isoleucine level and the second highest lysine value after WS70:STL30, while histidine was highest in the control. These patterns indicate that high STL inclusion was associated with greater essential amino acid abundance on a dry‐weight basis, whereas SCG mainly affected selected amino acids.

However, this compositional increase did not necessarily translate into improved protein quality. Chemical score calculations based on the adult reference amino acid pattern identified lysine as the limiting amino acid in the control and lower STL treatments, whereas histidine was limiting in WS70:STL30 and SCG‐supplemented treatments (Table S4). This pattern indicates that supplementation modified not only the total amino acid pool but also the proportional balance of indispensable amino acids. A substrate‐dependent response of amino acid composition has also been reported for *H. ulmarius* cultivated on Salicornia‐supplemented wheat straw, where supplementation affected the biochemical properties and amino acid profile of the fruiting bodies (Hausiku‐Ikechukwu et al. 2024). More broadly, previous studies on edible mushrooms indicate that the limiting amino acid is not fixed, but depends on mushroom species, processing conditions and the reference scoring pattern used. For instance, Jaworska and Bernaś (2013) reported that no limiting amino acid was found in fresh *Agaricus bisporus* under the FAO/WHO preschool‐child reference pattern, whereas lysine and leucine were identified as the first and second limiting amino acids, respectively, in fresh *Boletus edulis*. They also showed that freezing and pretreatments altered amino acid contents and chemical score values. In addition, methionine plus cystine was reported as the first limiting amino acid in *Morchella importuna* cultivated under different rotation systems (Su et al. 2022), tryptophan was limiting in several edible mushroom species from Thailand (Cuptapun et al. 2010), and no limiting amino acid was found in canned *Agaricus bisporus* and *Pleurotus ostreatus* when compared with FAO/WHO reference patterns (Jaworska and Bernaś 2011). Accordingly, the observed increase in total amino acid content should be interpreted as compositional enrichment rather than as confirmed improvement in protein quality. Because digestibility was not measured, PDCAAS or DIAAS could not be calculated, and the protein‐quality interpretation remains limited to chemical score comparisons.

A treatment‐dependent pattern was also observed in the non‐essential amino acid fraction. Arginine was the predominant amino acid in all treatments, followed by glycine, alanine, and glutamic acid. WS70:STL30 ranked among the top treatments for arginine, glutamic acid, glutamine, and cystine, whereas SCG formulations showed comparatively higher values for selected amino acids, including alanine, glycine, aspartic acid, and proline, depending on supplementation level. In contrast, tyrosine declined in SCG treatments relative to the control.

Similar substrate‐dependent variation in amino acid profiles has been reported in cultivated mushrooms Harada et al. (2004) showed that strain and cultivation medium affected soluble sugars and free amino acids in *Hypsizygus marmoreus*, and that supplementation of the cultivation medium improved MSG‐like taste components. Tagkouli et al. (2020) investigated free amino acids in *Pleurotus ostreatus*, *P. eryngii* , and *P. nebrodensis* cultivated on wheat straw, wheat straw mixed with grape marc, and olive‐industry by‐products, and reported significant differences in free amino acid levels among both species and substrates. Pellegrino et al. (2022) further showed, using LC/MS Q‐TOF metabolomic profiling, that amino acid and dipeptide profiles of *P. ostreatus* varied when the mushroom was grown on different substrates, including black poplar wood logs and lignocellulosic by‐products. More recently, Gardeli et al. (2024) reported that both strain and substrate influenced the amino acid composition and protein quality of *P. ostreatus* cultivated on wheat straw and barley–oat straw substrates. The present study extends these findings by showing that STL and SCG did not simply shift amino acid abundance in the same direction. High STL inclusion was associated with a broader increase in total amino acid content, whereas SCG caused more selective changes in individual amino acids. This may reflect differences in the nitrogenous composition, amino acid precursors, or degradability of the two residues, although this relationship requires further confirmation through direct linkage between substrate chemistry and fruiting‐body composition. From a food‐composition perspective, the STL response is notable because the increase in crude protein was also reflected in the amino acid profile. Nevertheless, the dietary relevance of these changes requires further evaluation using digestibility‐ or bioaccessibility‐based approaches.

### 3.5 Fatty Acid Composition

Several fatty acids differed among formulations based on relative peak‐area profiles in Table 6. Because fatty acids are expressed as percentages of total identified fatty acids, the results describe proportional redistribution within the lipid fraction rather than absolute fatty acid accumulation. Linoleic acid (C18:2 cis) remained the predominant fatty acid in all treatments, ranging from 15.78% in the control to 32.22%–49.68% in the supplemented groups. Oleic acid (C18:1) was the second major unsaturated fatty acid in supplemented treatments, ranging from 15.24% to 23.87%. Thus, supplementation did not alter the dominant fatty acid class but changed the relative distribution among major C18 fatty acids.

**TABLE 6:** Fatty acid composition of *Hypsizygus ulmarius cul*tivated under different growing medium formulations.

Component		Control	WS90:STL10	WS80:STL20	WS70:STL30	WS90:SCG10	WS80:SCG20	WS70:SCG30
Myristoleic A.	C14:1	1.72 ± 0.048	2.73 ± 0.023	3.24 ± 0.081	2.19 ± 0.097	1.69 ± 0.043	2.41 ± 0.028	2.06 ± 0.077
Palmitic A	C16:0	6.53 ± 0.018	9.45 ± 0.052	13.94 ± 0.217	10.46 ± 0.118	10.70 ± 0.276	11.75 ± 0.291	10.55 ± 0.459
Palmitoleic A.	C16:1	ND	ND	ND	ND	ND	ND	ND
Heptadecanoic A.	C17:0	3.93 ± 0.076	ND	ND	ND	ND	ND	ND
cis‐10‐heptadecanoic A.	C17:1	ND	ND	ND	ND	ND	ND	ND
Stearic A.	C18:0	4.26 ± 0.052	2.81 ± 0.044	5.83 ± 0.108	2.93 ± 0.065	3.18 ± 0.028	4.32 ± 0.059	3.49 ± 0.075
Oleic Acid	C18:1	9.20 ± 0.073	21.82	23.87 ± 0.311	15.24 ± 0.216	21.30 ± 0.287	16.17 ± 0.498	19.07 ± 0.769
Linoleic A.	C18:2 cis	15.78 ± 0.093	48.78 ± 0.118	32.22 ± 0.523	36.76 ± 0.498	46.46 ± 0.813	48.28 ± 0.749	49.68 ± 0.691
Arachidic A.	C20:0	2.16 ± 0.081	0.88 ± 0.004	0.67 ± 0.018	2.15 ± 0.077	1.08 ± 0.076	1.67 ± 0.087	2.76 ± 0.310
Linolenic A.	C18:3 (*n* − 3)	11.78 ± 0.089	3.57 ± 0.076	3.46 ± 0.076	3.37 ± 0.319	4.53 ± 0.345	3.04 ± 0.102	2.22 ± 0.211
Heneicosanoic A.	C21:0	5.49 ± 0.047	2.99 ± 0.071	0.88 ± 0.023	1.13 ± 0.065	0.51 ± 0.009	1.65 ± 0.045	1.26 ± 0.067
cis‐11,14‐eicosadienoic A.	C20:2	7.15 ± 0.069	ND	ND	2.49 ± 0.098	2.13 ± 0.210	2.23 ± 0.078	1.08 ± 0.091
Behenic A.	C22:0	4.57 ± 0.093	ND	1.06 ± 0.059	ND	ND	ND	ND
cis‐8,11,14‐eicosatrienoic A.	C20:3 (*n* − 3)	3.91 ± 0.072	ND	ND	ND	0.49 ± 0.035	0.96 ± 0.027	0.89 ± 0.006
Erucic A.	C22:1	2.61 ± 0.057	0.54 ± 0.038	1.28 ± 0.091	1.58 ± 0.045	0.34 ± 0.003	0.31 ± 0.008	ND
Tricosanoic A.	C23:0	3.35 ± 0.078	ND	ND	ND	ND	ND	ND
Lignoceric A.	C24:0	2.65 ± 0.043	1.68 ± 0.054	1.47 ± 0.116	1.70 ± 0.089	1.54 ± 0.023	1.17 ± 0.031	1.03 ± 0.061
cis‐13,16‐docosadienoic A.	C22:2	2.81 ± 0.041	ND	1.77 ± 0.098	ND	ND	ND	ND
cis‐5,8,11,14,17‐eicosapentaenoic A	C20:5	2.12 ± 0.018	0.68 ± 0.011	0.86 ± 0.056	0.36 ± 0.007	0.33 ± 0.005	0.53 ± 0.041	ND
Nervonic A.	C24:1	1.53 ± 0.009	0.54 ± 0.009	0.21 ± 0.007	0.17 ± 0.002	0.24 ± 0.009	1.05 ± 0.073	1.00 ± 0.059
cis‐4,7,10,13,16,19‐docosahexaenoic A.	C22:6	4.06 ± 0.039	ND	ND	0.52 ± 0.012	1.88 ± 0.041	3.08 ± 0.116	2.37 ± 0.129

*Note:* Value (%) = mean ± SD (*n* = 3).

Abbreviation: ND, not detectable.

The clearest formulation‐dependent contrast was observed between the SCG and STL series. In SCG‐supplemented treatments, linoleic acid remained consistently high (46.46%–49.68%), whereas STL treatments showed a wider range, from 32.22% in WS80:STL20 to 48.78% in WS90:STL10. Oleic acid partly contrasted this pattern within the STL series and reached its highest level in WS80:STL20 (23.87%). Palmitic acid (C16:0), the principal saturated fatty acid, increased from 6.53% in the control to 9.45%–13.94% in supplemented treatments, while stearic acid (C18:0) varied within a narrower range and reached its highest value in WS80:STL20 (5.83%). Linolenic acid [C18:3(*n* − 3)] declined from 11.78% in the control to 2.22%–4.53% in supplemented treatments. Minor long‐chain unsaturated fatty acids were detected only at low levels.

This pattern is consistent with previous reports showing that linoleic acid generally predominates in edible mushrooms, together with oleic, palmitic, and stearic acids as major lipid components (Günç Ergönül et al. 2013; Sande et al. 2019; Tan et al. 2024). Similar substrate‐dependent changes in mushroom fatty acid composition, including under spent coffee ground supplementation, have also been reported (Alsanad et al. 2021; Tan et al. 2024). The present findings indicate that post‐brewing residues can act as formulation variables that redirect lipid‐related compositional traits in *H. ulmarius*. SCG was associated with a consistently linoleic‐acid‐dominant profile, whereas STL showed broader variation in the relative balance between linoleic and oleic acids. This may reflect differences in residual lipid composition between SCG and STL or residue‐driven changes in fungal lipid metabolism, Given the treatment‐related differences in crude lipid content, absolute fatty acid concentrations expressed as mg/g DW would provide additional nutritional context by indicating whether the proportional shifts observed in the fatty acid profile correspond to actual changes in fatty acid content per unit dry mushroom biomass. Because the present fatty acid data were calculated from relative peak areas, they should be interpreted as proportional redistribution within the lipid fraction rather than direct evidence of absolute fatty acid accumulation.

### 3.6 FTIR Spectral Characteristics

**FIGURE 1:**
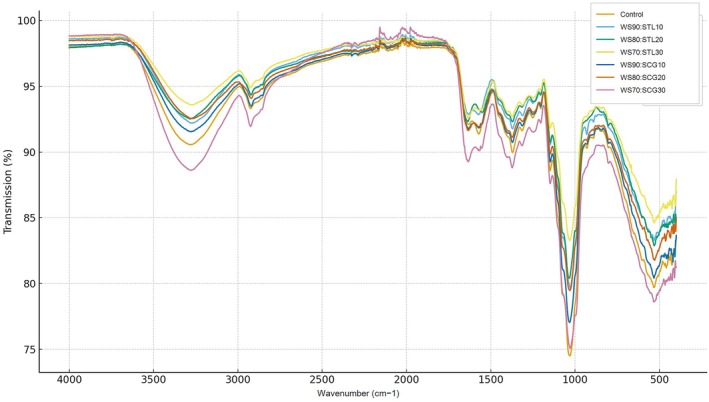
ATR‐FTIR spectra of *Hypsizygus ulmarius* fruiting bodies cultivated on control, STL‐supplemented, and SCG‐supplemented substrates.

FTIR spectra recorded over the 4000–500 cm^−1^ region showed a largely conserved spectral profile across all *H. ulmarius* fruiting body samples (Figure 1). The dominant absorptions were located in the carbohydrate‐associated fingerprint region (1200–900 cm^−1^), together with a band near 890 cm^−1^, while amide‐related bands, mainly amide I near 1650 cm^−1^ and amide II near 1545 cm^−1^, remained detectable in all treatments. This pattern suggests preservation of the glucan/chitin‐based fungal matrix across substrate formulations (Ruiz‐Herrera and Ortiz‐Castellanos 2019; Synytsya and Novak 2014). Within this conserved framework, qualitative differences were observed in selected spectral regions.

Bands associated with aliphatic C–H stretching at approximately 2920 and 2850 cm^−1^ and CH_3_ bending near 1375 cm^−1^ appeared more pronounced in SCG‐supplemented treatments. This observation is consistent with the higher crude lipid contents and lipid‐related compositional differences observed in these treatments (Tables 1 and 6), although FTIR does not allow direct identification or quantification of specific lipid classes. In contrast, the amide I region near 1650 cm^−1^ and amide II region near 1545 cm^−1^ appeared relatively more evident in selected STL treatments, particularly WS80:STL20 and WS70:STL30. These regions include overlapping contributions from proteins and chitin‐related structures and are therefore interpreted as protein/chitin‐associated features rather than specific markers of either component (Kumirska et al. 2010).

Additional qualitative variation was observed in the broad O–H/N–H stretching region at 3200–3500 cm^−1^ and within carbohydrate‐associated bands in the fingerprint region. These differences may reflect variation in hydrogen bonding, water association, and polysaccharide organization, although band overlap limits more specific interpretation From a food‐composition perspective, these spectral regions are relevant because they correspond to major macromolecular domains contributing to the structural and nutritional characteristics of edible mushrooms. The carbohydrate‐associated fingerprint region reflects the glucan/chitin‐based fungal matrix, while amide‐related and aliphatic C–H bands are associated with protein/chitin‐ and lipid‐related fractions, respectively (Kumirska et al. 2010; Synytsya and Novak 2014; Bekiaris et al. 2020). Therefore, the observed FTIR differences support the view that STL and SCG influenced food‐relevant matrix features of *H. ulmarius* fruiting bodies without substantially altering the overall fungal structural fingerprint.

The FTIR results provide supportive, qualitative evidence that substrate formulation was associated with changes in the relative spectral contributions of major macromolecular regions while maintaining the general fungal structural fingerprint. The observed spectral tendencies were consistent with lipid‐ and protein‐related compositional differences across treatments, but FTIR alone cannot confirm specific compound classes, quantify treatment effects, or establish direct correlations without complementary chemometric analysis such as band‐area ratios, second‐derivative analysis, or multivariate spectral modeling. Therefore, FTIR should be considered a complementary food‐characterization tool rather than a stand‐alone method for identifying treatment‐specific molecular changes.

### 3.7 Integrated Multivariate Analysis

Principal component analysis (PCA) was applied to integrate proximate composition, amino acid, fatty acid, mineral, and antioxidant variables and to assess whether the overall dataset supported residue‐specific differentiation of *H. ulmarius* fruiting bodies. FTIR data were not included in the multivariate analysis and were evaluated separately as complementary qualitative information.

**FIGURE 2:**
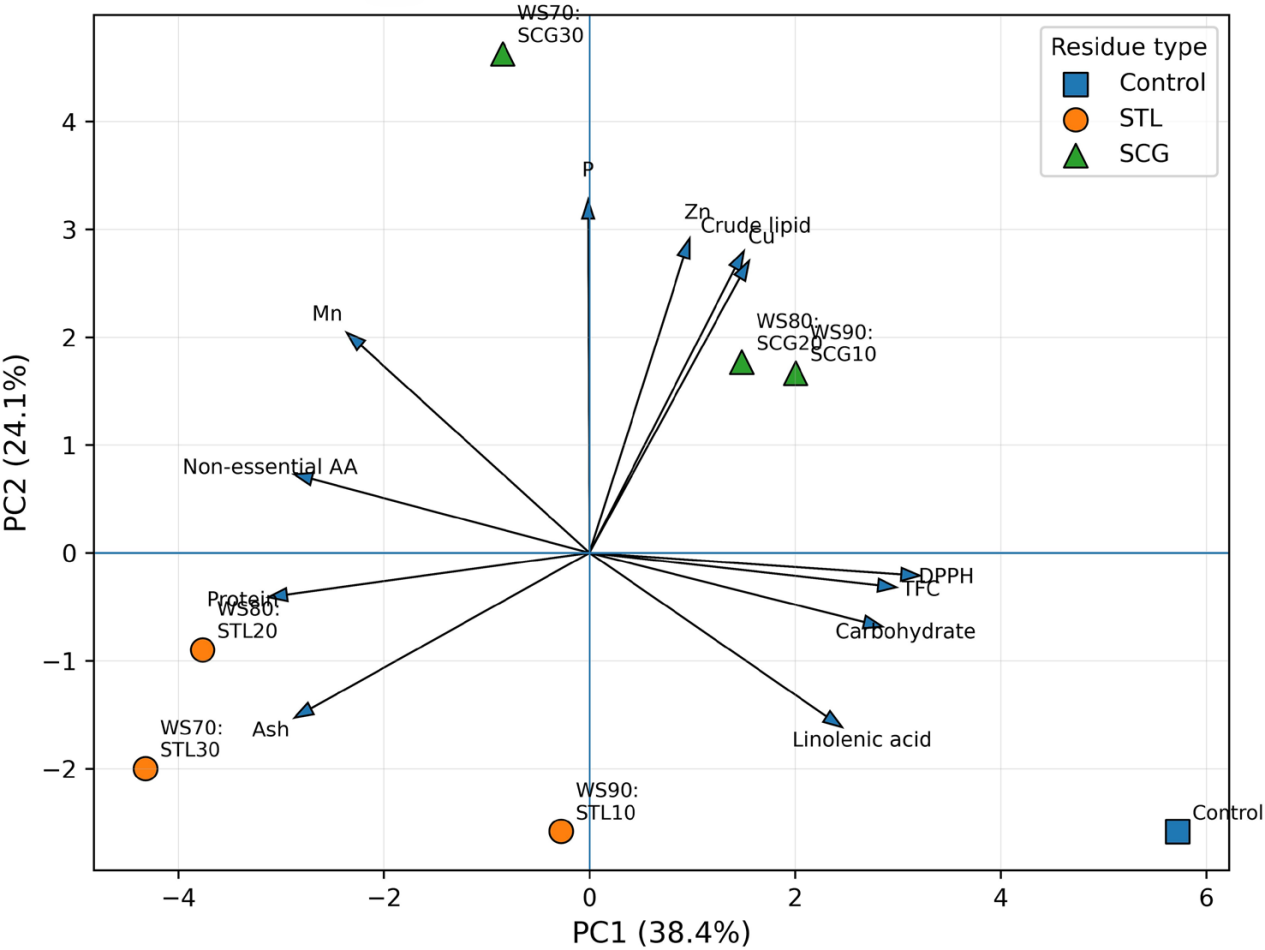
Principal component analysis biplot showing treatment‐related compositional differentiation of *Hypsizygus ulmarius* fruiting bodies cultivated on control, spent tea leaves (STL)‐supplemented, and spent coffee grounds (SCG)‐supplemented substrates.

The first two principal components explained 62.5% of the total variance, with PC1 and PC2 accounting for 38.4% and 24.1%, respectively (Figure 2). The score plot showed a distinct tendency toward separation between STL‐ and SCG‐based formulations, indicating that the two post‐brewing residues induced residue‐specific compositional response patterns rather than a uniform supplementation effect. The loading structure clarified the variables contributing to this differentiation. STL‐based treatments were mainly positioned on the negative side of PC1 and were more closely associated with protein and ash, while the non‐essential amino acid variable also loaded toward the STL side of the biplot. In contrast, SCG‐based treatments were positioned mainly on the positive side of PC1 and/or PC2 and showed closer associations with crude lipid and mineral‐related variables, particularly P, Zn, and Cu. Among the antioxidant‐related variables displayed in Figure 2, DPPH and TFC were oriented toward the positive side of PC1. These results suggest that residue type, rather than supplementation level alone, was an important factor shaping the overall compositional structure of *H. ulmarius* fruiting bodies.

## 4 Conclusion

This study demonstrates that STL and SCG function as distinct substrate formulation inputs for shaping the composition‐related quality attributes of *H. ulmarius* fruiting bodies. Among the STL treatments, WS70:STL30 was the most favorable formulation, producing the highest protein, ash, and total amino acid contents, together with the highest CUPRAC value. Among the SCG treatments, WS70:SCG30 was the most favorable formulation for lipid‐ and mineral‐related enrichment, particularly because it produced the highest crude lipid content and the highest concentrations of P, Mg, Zn, and Cu. Although the two residues improved different compositional domains, WS70:STL30 can be recommended as the most promising formulation when the main objective is to enhance the overall nutritional value of H. ulmarius fruiting bodies. In contrast, SCG supplementation may be more suitable when lipid‐related traits and selected mineral elements are targeted. These findings indicate that residue‐based mushroom cultivation can be positioned not only as a waste valorization strategy but also as a formulation approach for directing specific food‐compositional traits. Further studies integrating yield performance, bioaccessibility, sensory evaluation, storage behavior, and compound‐level validation are needed to determine the practical food relevance of these residue‐specific responses.

## Author Contributions

**Mehmet Çetin:** investigation, methodology, writing – review and editing, data curation, conceptualization, formal analysis, software. **Didem Aksu:** methodology, data curation, writing – review and editing, formal analysis. **Funda Atila:** conceptualization, investigation, writing – original draft, methodology, validation, visualization, software, formal analysis, project administration, data curation, supervision.

## Funding

This research was supported by the Research Fund of Ege University (Grant No. 32461).

## Conflicts of Interest

The authors declare no conflicts of interest.

## Supporting information


**Table S1:** (A) Proximate composition of spent tea leaves (STL) and spent coffee ground (SCG). (B) Total phenolic content and flavonoid content, antioxidant activity (ABTS, CUPRAC, DPPH), and total monomeric anthocyanin levels of spent tea leaves (STL) and spent coffee ground (SCG). (C) Macro and micro element contents of spent tea leaves (STL) and spent coffee ground (SCG).

**Table S2:** Gradient elution program used for amino acid analysis on the Agilent Eclipse AAA column.

**Table S3:** Mass fractions of all analytes quantified in the digest solutions of the reference material (SRM 1547) and recovery rates.

**Table S4:** Chemical amino acid scores and limiting amino acids of *H. ulmarius* fruiting bodies produced on STL‐ and SCG‐supplemented substrates.

## Data Availability

The data that supports the findings of this study are available in the Supporting Information of this article.
