# Supplementary material for: Nutritional and Compositional Profile of Hypsizygus ulmarius Fruiting Bodies as Affected by Spent Tea Leaves and Spent Coffee Grounds Supplementation
Source: Food Sci Nutr. 2026 Jul 23;14(7):e72141. doi: 10.1002/fsn3.72141 (PMC13392629; doi:10.1002/fsn3.72141)
Supplement: Supplementary file 1 — Table S1: (A) Proximate composition of spent tea leaves (STL) and spent coffee ground (SCG). (B) Total phenolic content and flavonoid content, antioxidant activity (ABTS, CUPRAC, DPPH), and total monomeric anthocyanin levels of spent tea leaves (STL) and spent coffee ground (SCG). (C) Macro and micro element contents of spent tea leaves (STL) and spent coffee ground (SCG). [file FSN3-14-e72141-s001.docx]

Table S1.A. Proximate composition of spent tea leaves (STL) and spent coffee ground (SCG)

| **Material** | **pH** | **EC**  **(mS/cm)** | **Ash**  **(g/100 g )** | **Carbon (g/100 g )** | **Nitrogen (g/100 g )** | **C:N** |
| --- | --- | --- | --- | --- | --- | --- |
| STL | 6.5±0.6 | 2.35±0,18 | 3.8±0.12 | 55.8±0.12 | 2.28±0.10 | 24.5±1.2 |
| SCG | 5.3±0.5 | 2.07±0.22 | 1.6±0.09 | 57.1±0.09 | 1.78±0.11 | 32.1±2.0 |

Values are expressed as mean ± standard deviation (n = 3)

Table S1.B. Total phenolic content and flavonoid content, antioxidant activity (ABTS, CUPRAC, DPPH), and total monomeric anthocyanin levels of spent tea leaves (STL) and spent coffee ground (SCG)

| **Material** | **Total Phenol**  **(mg GAE/g )** | **Total Flavonoid (mg QE/ g )** | **DPPH**  **(mM AAE/g dw )** | **CUPRAC**  **(µM AAS/g dw)** | **ABTS**  **(µmol TE/g dw )** | **Total Monomeric Anthocyanin (mg/L)** |
| --- | --- | --- | --- | --- | --- | --- |
| STL | 40.5±1.7 | 0.67±0,01 | 3.22±0.02 | 14.43±0.04 | 68.4±0.17 | 2.3±0.32 |
| SCG | 17.6±0.9 | 1.00±0,01 | 3.56±0.02 | 17.52±0.21 | 76.6±0.50 | 2.4±0.23 |

Values are expressed as mean ± standard deviation (n = 3)

Table S1.C. Macro and micro element contents of spent tea leaves (STL) and spent coffee ground (SCG)

| **Material** | **P**  **(mg/kg)** | **K**  **(mg/kg)** | **Ca**  **(mg/kg)** | **Mg**  **(mg/kg)** | **Na**  **(mg/kg)** | **Fe**  **(mg/kg)** | **Zn**  **(mg/kg)** | **Mn**  **(mg/kg)** | **Cu**  **(mg/kg)** |
| --- | --- | --- | --- | --- | --- | --- | --- | --- | --- |
| STL | 353.6±11.5 | 12126.4±28.4 | 5659.8±48.1 | 2358.7±52.6 | 1680.7±23.5 | 163.6±6.2 | 2.93±0.4 | 237.5±11.2 | 11.98±0.7 |
| SCG | 420.6±14.7 | 7881.0±31.7 | 3217.2±54.2 | 1763.9±71.4 | 938.6±18.4 | 106.8±7.4 | 3.29±0.2 | 138.8±16.1 | 12.37±0.3 |

Values are expressed as mean ± standard deviation (n = 3).
