# Supplementary material for: Nutritional and Compositional Profile of Hypsizygus ulmarius Fruiting Bodies as Affected by Spent Tea Leaves and Spent Coffee Grounds Supplementation
Source: Food Sci Nutr. 2026 Jul 23;14(7):e72141. doi: 10.1002/fsn3.72141 (PMC13392629; doi:10.1002/fsn3.72141)
Supplement: Supplementary file 2 — Table S2: Gradient elution program used for amino acid analysis on the Agilent Eclipse AAA column. [file FSN3-14-e72141-s003.docx]

Table S2. Gradient elution program used for amino acid analysis on the Agilent Eclipse AAA column.

| **Time (min)** | **Solvent A (%)** | **Solvent B (%)** |
| --- | --- | --- |
| 0.0 | 100 | 0 |
| 1.9 | 100 | 0 |
| 18.0 | 43 | 57 |
| 18.6 | 0 | 100 |
| 22.3 | 0 | 100 |
| 23.2 | 100 | 0 |
| 26.0 | 100 | 0 |

**Solvent A:** 40 mM borate buffer (pH 7.8)
**Solvent B:** acetonitrile:methanol:water (45:45:10, v/v/v)
