# Supplementary material for: Nutritional and Compositional Profile of Hypsizygus ulmarius Fruiting Bodies as Affected by Spent Tea Leaves and Spent Coffee Grounds Supplementation
Source: Food Sci Nutr. 2026 Jul 23;14(7):e72141. doi: 10.1002/fsn3.72141 (PMC13392629; doi:10.1002/fsn3.72141)
Supplement: Supplementary file 3 — Table S3: Mass fractions of all analytes quantified in the digest solutions of the reference material (SRM 1547) and recovery rates. [file FSN3-14-e72141-s004.docx]

Table S3. Mass fractions of all analytes quantified in the digest solutions of the reference material (SRM 1547) and recovery rates.

| **Element** | **Certified Value SRM 1547** | **Measured Value** | **Recovery (%)** | **CV (%)** |
| --- | --- | --- | --- | --- |
| P ( mg kg^-1^) | 1371 ± 82 | 1339.0±14.93 | 97.7 | 1.11 |
| K ( mg kg^-1^) | 24330 ± 380 | 23113.8±83.13 | 95.0 | 0.36 |
| Ca ( mg kg^-1^) | 15590 ± 160 | 15137.4±83.0 | 96.9 | 0.55 |
| Mg ( mg kg^-1^) | 4320 ± 150 | 4201.3±36.01 | 97.3 | 0.86 |
| Fe ( mg kg^-1^) | 219.8 ± 6.8 | 217.0±4.54 | 98.7 | 2.09 |
| Mn ( mg kg^-1^) | 97.8 ± 1.8 | 94.9±0.17 | 97.0 | 0.18 |
| Zn ( mg kg^-1^) | 17.97 ± 0.53 | 17.8±0.15 | 99.0 | 0.87 |
| Cu ( mg kg^-1^) | 3.75 ± 0.37 | 3.6±0.14 | 95.1 | 3.97 |
