# Supplementary material for: Nutritional and Compositional Profile of Hypsizygus ulmarius Fruiting Bodies as Affected by Spent Tea Leaves and Spent Coffee Grounds Supplementation
Source: Food Sci Nutr. 2026 Jul 23;14(7):e72141. doi: 10.1002/fsn3.72141 (PMC13392629; doi:10.1002/fsn3.72141)
Supplement: Supplementary file 4 — Table S4: Chemical amino acid scores and limiting amino acids of H. ulmarius fruiting bodies produced on STL‐ and SCG‐supplemented substrates. [file FSN3-14-e72141-s002.docx]

Table S4. Chemical amino acid scores and limiting amino acids of H. ulmarius fruiting bodies produced on STL- and SCG-supplemented substrates.

| **Treatment** | **Protein (g/100 g DW)** | **Total EAA (mg/g protein)** | **EAAI (%)** | **Limiting amino acid/group** | **Limiting score (%)** |
| --- | --- | --- | --- | --- | --- |
| Control | 14.63 | 50.13 | 21.89 | Lys | 10.13 |
| WS90:STL10 | 15.53 | 43.34 | 17.13 | Lys | 3.86 |
| WS80:STL20 | 16.77 | 43.07 | 17.73 | Lys | 5.52 |
| WS70:STL30 | 17.37 | 55.87 | 23.53 | His | 10.36 |
| WS90:SCG10 | 15.20 | 49.36 | 20.87 | His | 11.40 |
| WS80:SCG20 | 15.63 | 48.37 | 19.10 | His | 8.67 |
| WS70:SCG30 | 15.73 | 54.91 | 22.17 | His | 8.34 |

Note. Amino acid contents measured on a dry-weight basis (mg/100 g DW) were converted to mg/g protein using protein contents from proximate composition analysis. Chemical scores were calculated relative to the FAO/WHO/UNU adult indispensable amino acid scoring pattern: His 15, Ile 30, Leu 59, Lys 45, Met+Cys 22, Phe+Tyr 38, Thr 23, Trp 6, and Val 39 mg/g protein. Sulfur amino acids were calculated as Met+Cys, and aromatic amino acids as Phe+Tyr. EAAI, essential amino acid index. Chemical scores do not include digestibility; therefore, PDCAAS and DIAAS were not calculated.
